# Does Use of Low-Molecular-Weight Heparin during Pregnancy Influence the Risk of Prolonged Labor: A Population-Based Cohort Study

**DOI:** 10.1371/journal.pone.0140422

**Published:** 2015-10-14

**Authors:** Anna Sandström, Sven Cnattingius, Anna-Karin Wikström, Olof Stephansson, Anastasia N. Iliadou

**Affiliations:** 1 Clinical Epidemiology Unit, Department of Medicine Solna, Karolinska University Hospital, and Institutet, Stockholm, Sweden; 2 Department of Women’s and Children’s Health, Division of Obstetrics and Gynecology, Karolinska University Hospital, and Institutet, Stockholm, Sweden; 3 Department of Women’s and Children’s Health, Uppsala University, Uppsala, Sweden; 4 Department of Medical Epidemiology and Biostatistics, Karolinska Institutet, Stockholm, Sweden; Rutgers - New Jersey Medical School, UNITED STATES

## Abstract

**Background:**

The use of low-molecular-weight heparins (LMWHs) during pregnancy is increasing. *In vitro* studies and small clinical studies support the hypothesis that LMWH treatment during pregnancy may reduce duration of labor. The aim of this study was to investigate if use of LMWH is associated with a reduced risk of diagnosis of prolonged labor, after taking maternal, fetal and other delivery characteristics into account.

**Methods and Findings:**

A population-based cohort study from the Swedish Medical Birth Register from April 2006 through December 2011. We identified 514 875 term (≥37 weeks) deliveries of live singleton infants in cephalic presentation with spontaneous or induced onsets of labor. The Birth Register was linked to the Prescribed Drug Register to retrieve information on dispensed LMWH during pregnancy and to the Patient Register for information on underlying diagnosis for use of LMWH. Diagnosis of prolonged labor in the Birth Register was retrieved from diagnosis at discharge from the delivery hospital. The risk of diagnosis of prolonged labor in relation to treatment with LMWH was assessed using logistic regression analysis to estimate unadjusted and adjusted odds ratios. A total of 5 275 (1.0%) of the pregnant women used LMWH. The absolute risk of diagnosis of prolonged labor for nulliparous women was 19.9% among women using LMWH in third trimester, and 21.2% in women without use of LMWH. For parous women the corresponding absolute risks were 4.3% and 4.7%, respectively. Compared to nulliparous women without use of LMWH, nulliparous women with LMWH during third trimester had an odds ratio (OR) of 0.92 (95% CI 0.81–1.05, p-value: 0.051) for diagnosis of prolonged labor in unadjusted analyses and after adjustments for maternal characteristics, gestational age and epidural analgesia the OR was 1.00 (95% CI 0.87–1.15, p-value: 0.673). Parous women treated with LMWH in third trimester presented the same pattern, unadjusted OR for diagnosis of prolonged labor was 0.92 (95% CI 0.76–1.12, p-value: 0.418) and after adjustments OR was 0.99 (95% CI 0.80–1.22, p-value: 0.892). One limitation with the study was that information on prolonged labor was based on discharge diagnoses from the delivery hospital according to the International Classification of Diseases (ICD).

**Conclusions:**

Treatment with LMWH during pregnancy is not associated with a risk of diagnosis of prolonged labor after adjustments for maternal, fetal and delivery characteristics.

## Introduction

Prolonged labor, also known as labor dystocia, is a common clinical situation in obstetrical practice, and is associated with instrumental interventions and negative outcomes for both the mother and the infant [1–4]. There are several well-known factors associated with labor dystocia, including nulliparity, high maternal age, short maternal stature, high body mass index (BMI), post-term pregnancy, macrosomia (birth weight > 4500 g), labor induction and epidural analgesia [4–8]. However, the therapeutic arsenal to prevent or treat labor dystocia is limited. Oxytocin infusion is commonly used, but is associated with risks, notably fetal asphyxia [9,10]. New or complementary therapeutic alternatives would be appealing to provide as complement to existing treatment alternatives.

Low-molecular weight heparins (LMWHs) are increasingly used during pregnancy, either as prophylaxis for venous thromboembolism (VTE) due to the efficient reduction of the risk of VTE, or as an effective treatment of acute VTE in pregnancy, and are nowadays the drug of choice [11–14]. Other indications clinically practiced for LMWH use are recurrent miscarriages and prevention of placenta-mediated pregnancy complications.

In vitro studies have indicated that low-molecular weight heparins (LMWHs) increase both smooth muscle contractions in the uterus and cervical remodeling, which are two essential processes during normal labor [15]. About one percent of all pregnant women in Sweden use antithrombotic agents, including LMWHs, with a day-to-day or twice-a-day management [16]. Pregnancy is a strong independent risk factor for VTE and pregnancy-related VTE is a common cause for morbidity and one of the three most common obstetrical causes for maternal mortality in high income countries [17]. The cumulative incidence of pregnancy-related venous thromboembolism varies from 0.6–2.1/1 000 women in different populations [18–24]. The increased risk of VTE during pregnancy is observed already from the first trimester [18,20,25,26] where it is especially pronounced among women with assisted reproduction [27].

Systematic reviews have demonstrated the safety of LMWH, which neither cross the placenta, nor pass to breast milk [13,28]. There is less known about obstetric outcomes and complications; most studies are small and show contradictory results regarding risks of preterm delivery, fetal growth restriction, bleeding complications and cesarean section [11,29–36]. Two studies have demonstrated a shorter duration of labor for nulliparous women treated with the LMWH dalteparin and a reduced risk of prolonged first stage of labor [29,30]. These studies are relatively small and were not adjusted for potential confounders. The overall aim of our study was to investigate the hypothesis that use of LMWH during pregnancy is associated with a reduced risk of labor dystocia. This was examined in a large population-based cohort, taking maternal, delivery and fetal characteristics into account.

## Methods

### Register Data

**Fig 1:**
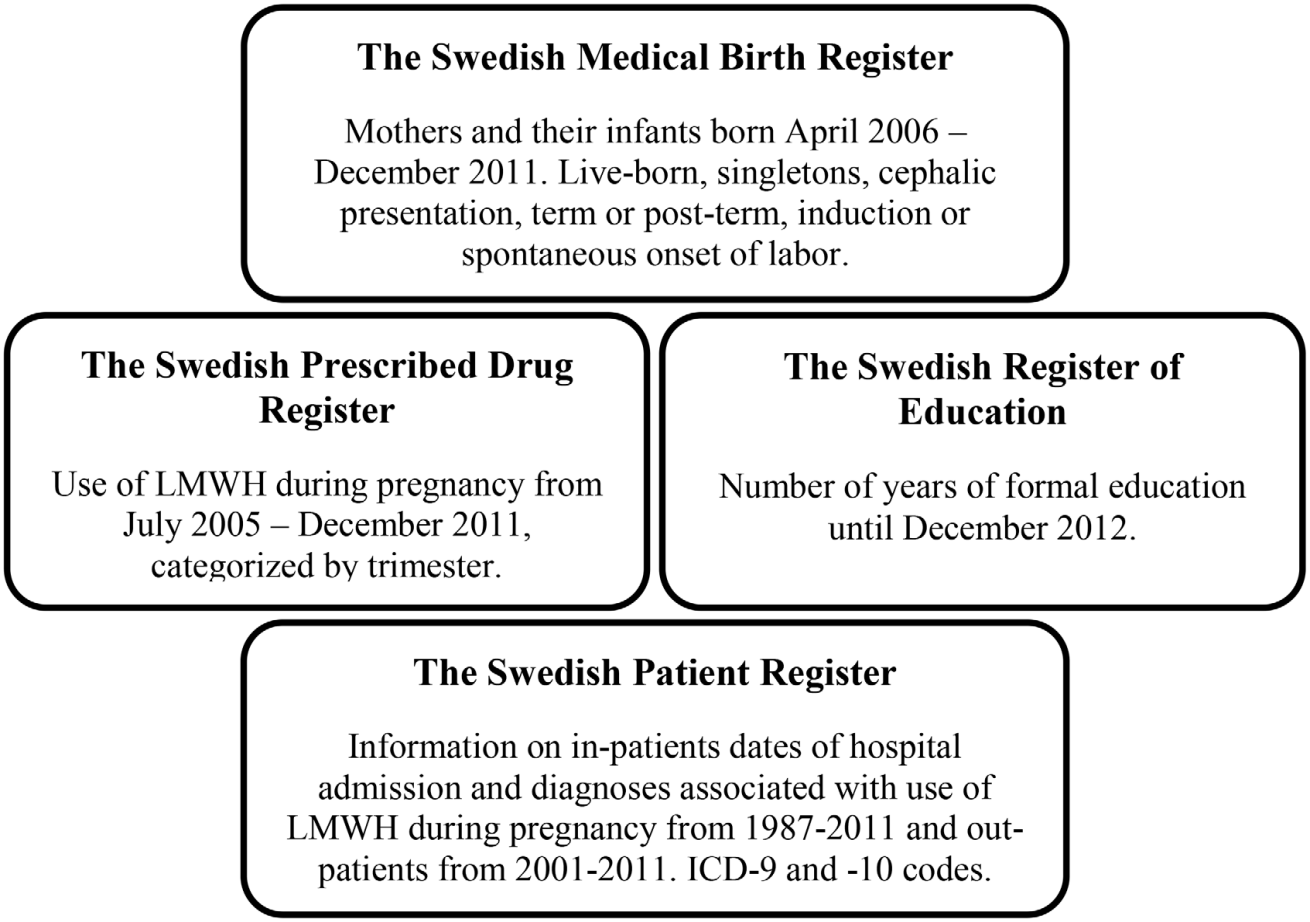
The Registers included in the study.

The study population in this cohort study was derived from the nation-wide Swedish Medical Birth Register (MBR) and was linked by individual record linkages to other national population based registers, using the person-unique identification number assigned to each citizen at birth or emigration to Sweden [37]. (Fig 1) The MBR includes more than 98% of all births in Sweden with prospectively collected information on demographic data, reproductive history and information from the pregnancy, delivery and neonatal period [38].

The Swedish Prescribed Drug Register started 1^st^ of July 2005, and includes data on the prescribed dispensed substance, brand name, ATC-code (Anatomical Therapeutic Chemical classification) and date of purchase for all dispensed drugs in the outpatient population [39].

The Swedish Patient Register, with information on in-patients, was established in 1964 (nationwide since 1987), and includes information on dates of hospital admissions, discharges, and diagnoses classified according to International Classification of Diseases (ICD), 7^th^ -10^th^ revisions (ICD-7 to ICD-10). Since 2001, the Registry also includes information on hospital out-patient visits [40].

The Swedish Register of Education is held by Statistics Sweden and contains information on the highest level of completed education of all Swedish citizens 16–74 years of age.

### Outcome variable

The outcome of the study was the diagnosis of labor dystocia. We used the ICD-10 codes in the MBR and the coding of diagnoses was made by attendant physicians at the delivery ward or at discharge. Diagnoses of labor dystocia included any of the following codes: primary (ICD-10 code: O62.0), and secondary dystocia (O62.1), prolonged first (O63.0) and second stage of labor (O63.1) and unspecified dystocia (O62.2, O62.9, O63.9 and O66.9). The code O62.2 includes both unspecified dystocia and prolonged latent phase. According to the Swedish version of ICD-10 codes and the Swedish Society for Obstetrics and Gynecology the following definitions are used: primary dystocia: dilation of the cervix less than 1 cm/hour during the active phase; secondary dystocia: no progress for at least 2 hours after initially normal progress; prolonged first stage of labor: a first stage of more than 15 hours in nulliparous women and 11 hours in parous women during the active phase; prolonged second stage of labor: a second stage of 2–3 hours or more for nulliparous women and 1–2 hours or more for parous women, the upper limit is modified by potential use of regional anesthesia.

### Exposure variables

Use of LMWH during pregnancy was the exposure. We used information on dispensed LMWHs, including dalteparin (ATC-code: B01AB04, Fragmin^®^), enoxaparin (B01AB05, Klexane^®^) and tinzaparin (B01AB10, Innohep^®^) from the Prescribed Drug Register. Exposure to any of the drugs were categorized into: a) before pregnancy (30–1 days before last menstrual period) and during pregnancy: b) first trimester (last menstrual period to 13 completed gestational weeks [i.e. to 13 weeks and 6 days]), c) second trimester (14 to 27 completed weeks) and d) third trimester (28 completed weeks or later).

We considered main exposure as ongoing use of LMWH during third trimester. However we could not á priori exclude the possibility that use during first and/or second trimester could have an effect on labor duration. We therefore analyzed use during first and/or second trimester as a secondary exposure.

Main exposure was thereby defined as use of LMWH during third trimester and included: exposure during first + second + third, first + third, second + third or third trimester only. Secondary exposure was LMWH during first and/or second trimester which included: exposure during only first, first + second or second trimester only. We also used information on exposure to warfarin (Waran^®^) during the period before pregnancy (30–1 days), because this is an indication for replacement with LMWH during pregnancy.

Specific doses are not adequately registered in the Prescribed Drug Register and could therefore not be investigated. According to Swedish recommendations, normal prophylaxis doses are 5000 U dalteparin or 4500 U tinzaparin or 40 mg enoxaparin once daily in women with early pregnancy weight 90 kg or lower, and higher initial doses in overweight women guided by anti-FXa-activity (3 hours after injection 10–14 days after start of the treatment, a level of 0.2–0.45 U/ml). High dose prophylaxis is administrated twice daily and is guided by measurable anti-FXa-activity level 12-hours (i.e. a level of 0.1–0.2 U/ml before next injection), the initial dalteparin dose among women with early pregnancy weight 50–80 kg is usually 5000 units twice daily, tinzaparin 175 U/kg and enoxaparin 40 mg twice daily [41,42]. Treatment dose for deep vein thrombosis for dalteparin is 125 U/kg twice daily and thereafter guided by the anti-FXa-activity (Anti-FXa-activity before injection 0.2–0.3 U/ml and after 4–6 weeks a reduced dose with an activity corresponding to 0.1–0.2 U/ml) [41].

### Underlying diagnoses

We recorded diagnoses associated with LMWH treatment from the Patient Register. Information on in-patient diagnoses from 1987 to 2011 (ICD: 9–10) and outpatient diagnoses from 2001 to 2011 (ICD-10) were divided into ten groups: 1) Deep venous thrombosis, 2) cerebral venous thrombosis, 3) pulmonary embolus, 4) other thromboses/emboli, 5) atrial fibrillation, 6) mechanical heart valve, 7) primary thrombophilia (including heterozygous or homozygous factor V Leiden-mutation, deficiency of antithrombin, protein C or protein S, prothrombin gene mutation), 8) other thrombophilia (including antiphospholipid syndrome, anticardiolipin syndrome, and presence of lupus anticoagulant), 9) infertility and 10) recurrent pregnancy loss (three or more consecutive spontaneous abortions). For ICD-9 and -10 codes, see S1 Table. We used information on dates of the diagnoses to group them into before or during pregnancy. The woman can have more than one of the ten groups of diagnoses, but the same group of diagnoses (1 to 10) could only be recorded once before and once during the pregnancy. It is noteworthy that diagnoses for each pregnancy are recorded, i.e. the same woman can be represented several times, with the same or additional diagnoses recorded in following deliveries.

We classified women with use of LMWH into presumed prophylactic dose or presumed high dose based on the underlying diagnoses according to current Swedish recommendations [42,43]. The presumed high dose group included both indications for high dose prophylaxis and for treatment dose. This included women with diagnoses of thrombosis during present pregnancy, previous and present thromboses, antiphospholipid syndrome with previous or present thromboses, mechanical heart valve and continuous warfarin or LMWH treatment before the pregnancy (30 days). Diagnoses of repeated thromboses before pregnancy and antithrombin deficiency are also indications for high dose prophylaxis but were not possible to include. The remaining women with LMWH treatment were considered having presumed prophylactic dose.

### Variables

From MBR we retrieved information about parity and classified the women as nulliparous or parous. Maternal age at delivery was categorized into four groups: ≤ 29, 30–34, 35–39 and ≥ 40 years. Information on maternal height and body mass index (BMI) was collected at the first attendance to antenatal care in early pregnancy. Maternal height was categorized in four groups: ≤ 154, 155–164, 165–174 and ≥ 175 cm. BMI (kg/m^2^) was divided in groups according to the World Health Organization as underweight (< 18.5), normal weight (18.5–24.9), overweight (25.0–29.9), obese class I (30.0–34.9) and obese class II and III (≥ 35) [44]. Self-reported information on cigarette smoking during pregnancy was categorized into yes (including daily smoking in the beginning of the pregnancy and/or in gestational weeks 30–32) and no (reporting no current smoking).

Information on hypertensive diseases and diabetes was also retrieved from MBR, which holds information on concurrent diseases reported as check boxes from the standardized antenatal care record or from maternal diagnosis with ICD-10 codes at discharge from the delivery hospital. Hypertensive disease was categorized as no hypertension, chronic hypertension, and preeclampsia, and diabetes as no diabetes, pre-gestational (Type I and II) diabetes, and gestational diabetes.

The standardized antenatal record also contains information on assisted reproductive technology, categorized into no treatment, in vitro fertilization (including intracytoplasmatic sperm injection) and ovulation induction.

Gestational length was based on ultrasound examination for more than 95% of the pregnancies, usually between 17–19 gestational weeks [45]. If information on ultrasound examination was missing, last menstrual period was used for dating of pregnancy. Gestational length at delivery was categorized into completed weeks, from 37 to ≥42 weeks. Information on onset of delivery (induction or spontaneous), use of epidural analgesia and infant birth weight, were retrieved from the standardized delivery records. Birth weight was categorized into: <3 000, 3 000–3 499, 3 500–3 999, 4 000–4 499 and ≥4 500 grams. The Education Register includes information on number of years of completed formal education as of 31^st^ of December 2012. Information was categorized as ≤ 12 years and > 12 years of formal education.

### Study population

**Fig 2:**
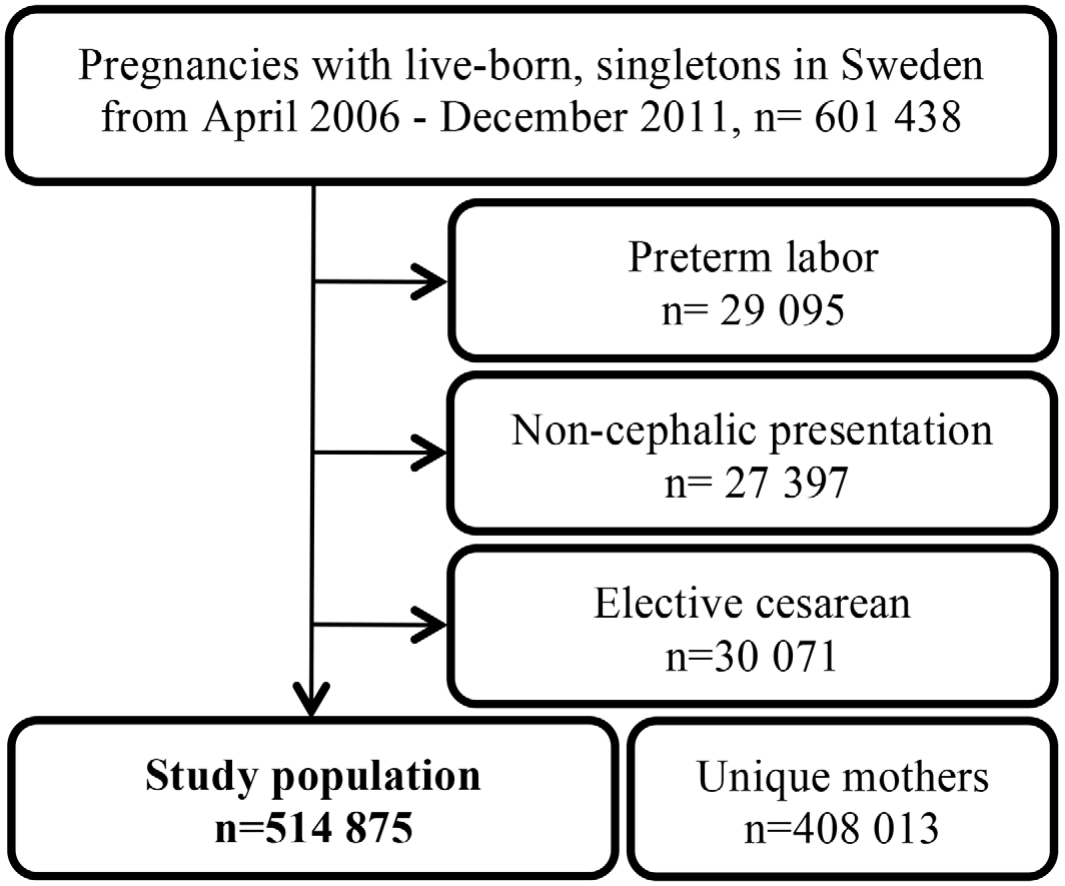
Flowchart of the study population.

From the MBR we created a cohort of deliveries with live singleton infants born between April 1^st^, 2006 and December 31^st^, 2011. After excluding preterm births, infants born in non-cephalic presentation and elective cesarean deliveries, the study population consisted of 514 875 deliveries with 408 013 unique mothers. The study period started April 1^st^, 2006 due to the beginning of the drug register in 1^st^ of July 2005, enabling the mothers to be exposed to LMWH throughout pregnancy. (Fig 2) All data was anonymized and de-identified prior to analysis.

### Statistical analyses

We estimated the risk of labor dystocia in relation to use of LMWH during pregnancy by unadjusted and adjusted odds ratios (ORs) with 95% confidence interval (CI). All analyses were stratified into nulliparous or parous women. In adjusted models, possible confounders were taken into account, including maternal characteristics (maternal age, height, BMI, cigarette smoking, diabetes, hypertensive disease, assisted reproduction, and years of formal education) and possible delivery confounders (gestational age, onset of delivery, epidural analgesia, infant birth weight, and calendar year of birth). Generalized estimated equations were used for the logistic regression analysis in order to account for the correlation between dependent observations (mothers with more than one child in the study population). Test for interaction was done in the logistic regression model to estimate the possible effect modification by onset of labor (spontaneous or induction) and epidural analgesia on the association between use of LMWHs and labor dystocia. Stratified analyses for epidural analgesia were also conducted. In additional analyses we compared women with presumed high dose and presumed prophylactic dose of LMWH with women without treatment. Data were analyzed using the SAS software version 9.2.

## Results

In the study population of 514 875 deliveries, 45.1% of the deliveries were by nulliparous and 54.9% by parous women (Table 1). In total, 5 275 deliveries (1.0%), were exposed to LMWH during pregnancy, 2 129 (0.9%) among nulliparous women and 3 146 (1.1%) among parous women. The absolute risk of labor dystocia was 21.2% for nulliparous women and 4.7% for parous women. Of women exposed to LMWH during pregnancy, dalteparin was used by 79.4%, tinzaparin by 21.4% and enoxaparin by 1.6% (131 women used two out of the three LMWHs during pregnancy).

**Table 1:** Maternal, fetal and delivery characteristics and diagnosis of labor dystocia in nulliparous and parous women with singleton infants in cephalic presentation, term or post-term births with induction or spontaneous onset of delivery, in Sweden, April 2006-December 2011.

	Nulliparous women				Parous women			
		Labor dystocia				Labor dystocia		
Maternal characteristics	N total	%	OR	(95% CI)	N total	%	OR	(95% CI)
**Labor dystocia**	232 104	21.2			282 771	4.7		
**Use of LMWH**								
No use*	229 975	21.2	1.00	(-)	279 625	4.7	1.00	(-)
Third trimester	1 442	19.9	0.92	(0.81–1.05)	2 418	4.3	0.92	(0.76–1.12)
First and/or second trimester	687	23.9	1.17	(0.98–1.39)	728	5.5	1.19	(0.87–1.64)
**Age (years)**								
≤ 29*	139 307	18.3	1.00	(-)	91 152	4.0	1.00	(-)
30–34	67 135	24.3	1.43	(1.40–1.47)	111 556	4.7	1.17	(1.12–1.22)
35–39	21 972	28.7	1.80	(1.74–1.86)	66 404	5.2	1.33	(1.27–1.39)
≥ 40	3 690	29.6	1.88	(1.75–2.02)	13 659	6.0	1.53	(1.42–1.66)
**Height (cm)**								
≤ 154	6 384	27.8	1.55	(1.46–1.64)	8 771	7.6	1.95	(1.80–2.12)
155–164	75 714	23.6	1.24	(1.22–1.27)	95 134	5.7	1.42	(1.37–1.48)
165–174*	115 347	19.9	1.00	(-)	139 039	4.1	1.00	(-)
≥ 175	23 929	17.6	0.86	(0.83–0.89)	27 176	3.1	0.76	(0.71–0.82)
Missing	10 730				12 651			
**BMI (kg/m** ^**2**^ **)**								
< 18.5	6 113	16.3	0.77	(0.72–0.82)	4 984	2.9	0.70	(0.59–0.83)
18.5–24.9*	139 130	20.3	1.00	(-)	150 881	4.1	1.00	(-)
25.0–29.9	48 270	23.0	1.18	(1.15–1.21)	70 628	5.3	1.31	(1.26–1.37)
30.0–34.9	14 435	24.1	1.25	(1.20–1.31)	24 427	5.9	1.49	(1.41–1.58)
≥ 35	5 821	25.8	1.37	(1.29–1.45)	10 064	7.1	1.81	(1.67–1.96)
Missing	18 335				21 787			
**Smoking during pregnancy**								
No*	207 649	21.5	1.00	(-)	253 030	4.7	1.00	(-)
Yes	16 435	17.1	0.75	(0.72–0.78)	20 341	4.3	0.92	(0.85–0.98)
Missing	8 020				9 400			
**Diabetes**								
No*	229 461	21.1	1.00	(-)	278 690	4.6	1.00	(-)
Gestational	1 924	25.3	1.26	(1.14–1.40)	3 171	6.5	1.44	(1.25–1.66)
Pre-gestational	719	27.8	1.44	(1.22–1.69)	910	6.8	1.51	(1.17–1.95)
**Hypertensive disease**								
No*	222 954	21.2	1.00	(-)	277 475	4.6	1.00	(-)
Chronic	1 293	24.2	1.19	(1.05–1.35)	1 906	5.7	1.25	(1.03–1.52)
Preeclampsia	7 857	21.0	0.99	(0.94–1.05)	3 390	6.2	1.36	(1.19–1.57)
**Assisted reproduction**								
No*	217 422	21.1	1.00	(-)	275 769	4.6	1.00	(-)
*In vitro* fertilization	10 674	23.7	1.17	(1.11–1.22)	4 901	5.7	1.24	(1.10–1.40)
Ovulation stimulation	4 008	22.2	1.07	(0.99–1.15)	2 101	5.8	1.26	(1.05–1.52)
**Education**								
Years of formal education ≤12*	102 314	20.3	1.00	(-)	138 447	4.8	1.00	(-)
Years of formal education >12	123 417	22.1	1.12	(1.09–1.14)	137 956	4.5	0.95	(0.92–0.98)
Missing	6 373				6 368			
**Fetal and delivery characteristics**								
**Gestational length at birth (weeks)**								
37	10 807	12.7	0.54	(0.51–0.57)	12 363	3.2	0.66	(0.59–0.73)
38	24 943	15.0	0.65	(0.63–0.68)	33 908	3.2	0.66	(0.62–0.70)
39	52 468	17.2	0.77	(0.75–0.79)	72 345	3.7	0.76	(0.73–0.80)
40*	71 508	21.3	1.00	(-)	92 468	4.8	1.00	(-)
41	50 375	26.2	1.31	(1.27–1.34)	54 455	6.1	1.30	(1.24–1.36)
≥ 42	21 927	30.1	1.59	(1.53–1.64)	17 102	7.5	1.62	(1.52–1.73)
Missing	76				130			
**Onset of delivery**								
Spontaneous*	197 122	20.3	1.00	(-)	246 334	4.3	1.00	(-)
Induction	34 057	26.5	1.41	(1.38–1.45)	35 188	7.0	1.67	(1.60–1.75)
Missing	925				1 249			
**Epidural analgesia**								
No*	115 960	11.2	1.00	(-)	230 390	2.6	1.00	(-)
Yes	116 144	31.1	3.57	(3.50–3.65)	52 381	13.8	6.02	(5.81–6.24)
**Birth weight (grams)**								
< 3000	28 932	11.5	0.64	(0.61–0.67)	21 000	2.8	0.81	(0.74–0.89)
3000–3499*	85 649	16.9	1.00	(-)	83 197	3.4	1.00	(-)
3500–3999	82 233	23.6	1.52	(1.48–1.56)	110 473	4.5	1.34	(1.27–1.40)
4000–4499	29 339	32.5	2.37	(2.30–2.44)	54 054	6.4	1.96	(1.86–2.06)
≥4500	5 624	41.8	3.53	(3.34–3.73)	13 747	9.3	2.90	(2.71–3.11)
Missing	327				300			

* Reference.

The rates of labor dystocia for nulliparous women were 21.2% for those with no use of LMWH, 19.9% for those using LMWH in third trimester, and 23.9% for those using LMWH in first and/or second trimester (Table 1). For parous women, the corresponding rates were 4.7%, 4.3%, and 5.5%, respectively. Analyzing the three LMWH:s dalteparin, tinzaparin and enoxaparin separately in third trimester, demonstrated rates of labor dystocia for nulliparous women of 20.4%, 17.9% and 23.8% respectively. Among women using LMWH in first and/or second trimester, a substantial proportion conceived with in vitro fertilization (45.6% among nulliparous and 14.8% among parous women).

In unadjusted models, use of LMWH was not associated with labor dystocia among nulliparous or parous women. In contrast, increasing age, short maternal stature, higher BMI, diabetes (gestational and pre-gestational), chronic hypertension and in vitro fertilization were all significantly associated with labor dystocia in both nulliparous and parous women. Smokers had a reduced risk of labor dystocia. Risk of labor dystocia also increased with increasing gestational length and was higher following induction than after spontaneous onset of labor. Labor dystocia was strongly associated with epidural analgesia, especially in parous women, and with increasing birth weight, especially in nulliparous women (Table 1). Information on LMWH use by trimester and maternal characteristics is provided in S4 Table.

For nulliparous women, epidural analgesia was used in 41.5% of those with LMWH in third trimester and in 50.1% of those without LMWH in third trimester (p-value < 0.0001). For parous women, corresponding rates were 16.3% and 18.5%, respectively (p-value 0.0039).

Table 2 demonstrates underlying diagnoses, before (from 1987 and onwards) and during pregnancy in women using LMWH. Deep venous thrombosis was the most common underlying diagnosis for treatment. Pulmonary embolus, primary thrombophilia and other thromboses/emboli were also common underlying diagnoses. Infertility was common among women with LMWH use in first and/or second trimester.

**Table 2:** Use of LMWH during pregnancy and eligible underlying diagnoses.

	Use of LMWH			
	Nulliparous women		Parous women	
	(N = 2 129)		(N = 3 146)	
Diagnoses before and/or during pregnancy	Third trim	First and/or second trim	Third trim	First and/or second trim
	(N = 1 442)	(N = 687)	(N = 2 418)	(N = 728)
**Deep venous thrombosis**	506	40	891	108
**Cerebral venous thrombosis**	23	2	39	8
**Pulmonary embolus**	184	12	336	33
**Other thromboses/emboli**	154	17	248	32
**Atrial fibrillation**	3	0	11	2
**Mechanical heart valve**	4	0	5	0
**Primary thrombophilia** *	154	20	238	37
**Other thrombophilia** **	25	4	38	4
**Infertility**	194	373	197	174
**Recurrent pregnancy loss** ***	92	58	112	117
**Others**	478	197	873	311

* Including factor V Leiden-mutation (heterozygous and homozygous), deficiency of antithrombin, protein C or protein S, prothrombin gene mutation, other primary thrombophilia.

** Including antiphospholipid syndrome, anticardiolipin syndrome and presence of lupus anticoagulant, other specified thrombophilia.

***Three or more consecutive spontaneous abortions

**Fig 3:**
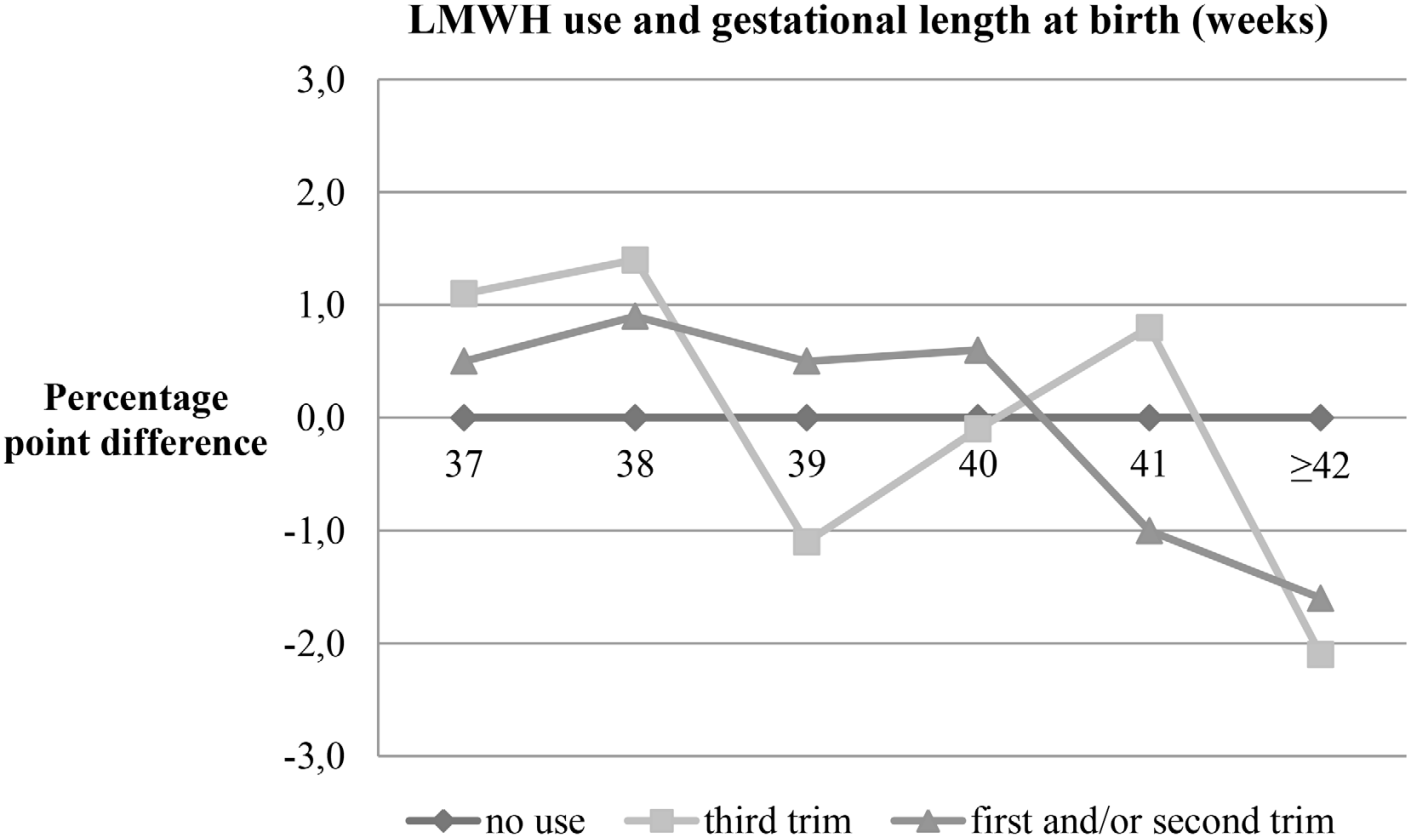
Gestational length at birth (weeks) and use of LMWH during pregnancy, all deliveries.

Fig 3 illustrates the association between gestational length at birth and use of LMWH during third or first and/or second trimester compared with no use. There seems to be no clear relation between gestational length at birth and use of LMWH in third trimester. However, the proportion of women with LMWH in first and/or second trimester compared to no use decrease after gestational week 40 with approximately 1 percent point difference per week.

Table 3 illustrates the risk of diagnosis of labor dystocia in women treated with LMWH in third trimester or in first and/or second trimester compared to no treatment. For nulliparous women, LMWH treatment during third trimester was, if anything, associated with a slightly reduced risk of labor dystocia after adjusting for maternal characteristics (OR 0.87 [95% CI 0.76–1.00]) (model 1). When we also introduced gestational length at birth and epidural analgesia in the adjusted analysis, there was no effect of LMWH use on labor dystocia (OR 1.00 [95% CI 0.87–1.15]) (model 3). Including birth weight in the model did not substantially influence the results (model 4). The analyses for parous women treated with LMWH in third trimester compared to no treatment illustrated the same pattern (Table 3).

**Table 3:** Regression analysis of use of LMWH during pregnancy and diagnosis of labor dystocia in nulliparous and parous women with singleton infants in cephalic presentation, term or post-term births with induction or spontaneous onset of delivery, in Sweden, April 2006 -December 2011.

	Labor dystocia							
	**Nulliparous women**							
	**(N = 232 104)**							
**Use of LMWH**	**aOR** * **mod 1**	**(95% CI)**	**aOR** ** **mod 2**	**(95% CI)**	**aOR** *** **mod 3**	**(95% CI)**	**aOR** **** **mod 4**	**(95% CI)**
**No use**	1.00	(-)	1.00	(-)	1.00	(-)	1.00	(-)
**Third trimester**	0.87	(0.76–1.00)	0.90	(0.79–1.04)	1.00	(0.87–1.15)	1.03	(0.89–1.19)
**First and /or second trimester**	1.01	(0.84–1.22)	1.04	(0.87–1.26)	1.08	(0.89–1.31)	1.09	(0.89–1.32)
	**Parous women**							
	**(N = 282 771)**							
**No use**	1.00	(-)	1.00	(-)	1.00	(-)	1.00	(-)
**Third trimester**	0.85	(0.69–1.05)	0.89	(0.72–1.10)	0.99	(0.80–1.22)	1.03	(0.83–1.27)
**First and /or second trimester**	0.97	(0.68–1.37)	1.03	(0.73–1.46)	0.96	(0.68–1.38)	0.99	(0.69–1.42)

* Model 1: Adjustments for maternal characteristics: treatment with LMWH, age, height, BMI, smoking during pregnancy, diabetes, hypertensive disease, assisted reproduction, education, year of birth and onset of labor.

** Model 2: Adjustment for characteristics in model 1, and gestational length at birth.

*** Model 3: Adjustment for characteristics in model 1, gestational length at birth and epidural analgesia.

**** Model 4: Adjustment for characteristics in model 1, gestational length at birth, epidural analgesia and birth weight.

S2 and S3 Tables present absolute and relative risks in unadjusted and adjusted analyses for LMWH treatment and dystocia stratified by epidural analgesia. The absolute risks of dystocia were higher in all groups of women with epidural analgesia compared to no epidural analgesia. The adjusted odds ratios for treatment with LMWH compared to no treatment did not show any statistically significant effect, and were similar for nulliparous and parous women with and without epidural (S2 and S3 Tables).

In Table 4, women treated with LMWH in third trimester were divided into presumed prophylactic dose or presumed high dose. Compared to no treatment, nulliparous women with presumed prophylactic dose had a reduced risk of labor dystocia after adjustments for maternal characteristics, OR 0.83 (95% CI 0.71–0.97). However, after further adjustments in models 2–4, this risk was no longer significantly reduced. In nulliparous women, there was no association between presumed high dose LMWH and labor dystocia in the unadjusted model, but in models 3 and 4, presumed high dose was associated with increased risk of labor dystocia, OR 1.41 (95% CI 1.06–1.86) and 1.48 (95% CI 1.12–1.97), respectively (Table 4).

**Table 4:** Use of LMWH, divided by presumed high dose and presumed prophylactic dose in third trimester compared to no treatment, and labor dystocia. Women with treatment in first and/or second trimester are excluded.

	Labor dystocia											
	**Nulliparous women**											
	**(N = 231 417)**											
	**N Total**	**Labor dystocia (%)**	**OR**	**(95% CI)**	**aOR** * **mod 1**	**(95% CI)**	**aOR** ** **mod 2**	**(95% CI)**	**aOR** *** **mod 3**	**(95% CI)**	**aOR** **** **mod 4**	**(95% CI)**
**No use**	229 975	21.2	1.00	(-)	1.00	(-)	1.00	(-)	1.00	(-)	1.00	(-)
**Presumed prophylactic dose**	1 096	19.3	0.89	(0.76–1.03)	0.83	(0.71–0.97)	0.85	(0.73–1.00)	0.90	(0.77–1.06)	0.92	(0.78–1.09)
**Presumed high dose** *****	346	22.0	1.05	(0.81–1.35)	1.02	(0.77–1.33)	1.08	(0.82–1.42)	1.41	(1.06–1.86)	1.48	(1.12–1.97)
	**Parous women**											
	**(N = 282 043)**											
**No use**	279 625	4.7	1.00	(-)	1.00	(-)	1.00	(-)	1.00	(-)	1.00	(-)
**Presumed prophylactic dose**	1 979	4.7	1.00	(0.81–1.23)	0.93	(0.74–1.16)	0.97	(0.78–1.21)	1.05	(0.84–1.32)	1.09	(0.87–1.37)
**Presumed high dose** *****	439	2.7	0.58	(0.32–1.02)	0.51	(0.28–0.94)	0.54	(0.30–0.99)	0.67	(0.36–1.23)	0.70	(0.38–1.29)

***** The presumed high dose group is based on underlying diagnoses with indications for treatment dose and high dose prophylaxis: thrombosis during present pregnancy, previous and present thrombosis, antiphospholipidantibody syndrome with previous or present thrombosis, mechanical heart valve, and continuous warfarin or LMWH treatment before the pregnancy (30 days). Previous several thrombosis and antithrombin deficiency were not included. The presumed prophylactic group includes the residual women with LMWH treatment in third trimester.

* Model 1: Adjustments for maternal characteristics: treatment with LMWH, age, height, BMI, smoking during pregnancy, diabetes, hypertensive disease, assisted reproduction, education, year of birth and onset of labor.

** Model 2: Adjustment for characteristics in model, and gestational length at birth.

*** Model 3: Adjustment for characteristics in model 1, gestational length at birth and epidural analgesia.

**** Model 4: Adjustment for characteristics in model 1, gestational length at birth, epidural analgesia and birth weight.

In the presumed high dose group for parous women (only 12 observations), there was a reduced risk of labor dystocia in unadjusted analyses and in adjusted models 1 and 2 (model 2: OR 0.54 (95% CI 0.30–0.99)). After further adjustments in models 3–4, this finding was no longer statistically significant (Table 4).

## Discussion

In this population-based cohort study of more than 500 000 births, use of LMWH in third trimester among both nulliparous and parous women was not associated with labor dystocia after adjustments for maternal, delivery and fetal characteristics.

A major strength of this study is the population-based design, including almost all deliveries in Sweden from April 2006 to December 2011. As a result of the large size of the study and due to national register linkages, we were able to analyze the results stratified by parity, epidural use, and treatment period during pregnancy, and we were also able to adjust for several important potential confounders. All information during maternal care and delivery were prospectively collected excluding the possibility of recall bias. Information on underlying diagnoses for LMWH use during pregnancy was also available.

To our knowledge, the effect of LMWH on duration of labor has only been investigated in two clinical studies [29,30]. Isma et al. found that 104 nulliparous women exposed to the LMWH dalteparin had, compared to 787 untreated women, one hour shorter first stage of labor and a significantly lower risk of prolonged first stage of labor (4.1% vs 8.5%, respectively P = 0.047). No such relationships were found for parous women [29]. One of the main differences with our study is that we examined the diagnosis of labor dystocia and not the actual duration of labor. Further, in the study by Isma et al., there were significant differences for confounders as maternal age, maternal weight, epidural analgesia, gestational length at delivery and frequency of preterm deliveries in the exposed and unexposed groups. This was not accounted for in the analysis and may partly explain the shorter duration of labor among women with use of LMWH [29].

In the study by Ekman-Ordeberg et al., 99 nulliparous women treated with dalteparin had shorter mean labor duration than women in the unexposed control group, 6 vs. 9 hours respectively. In contrast also to the study by Ekman-Ordeberg et al. we examined the diagnosis of dystocia instead of the total labor duration. Other differences to the study by Ekman-Ordeberg et al. were that we included women with emergency cesarean deliveries, where labor dystocia is one of the main indications and that we included women with induction of labor, which was more common among LMWH users. Induced women are also more prone to suffer from dystocia [4]. In our study, onset of labor (spontaneous or induction) was not found to be an effect modifier on the association between use of LMWHs and labor dystocia, and could thereby be treated as a confounder. In the study by Ekman-Ordeberg et al., exposed and unexposed nulliparous women were age matched, and stratified analyses were made for use of epidural analgesia, however, no adjustments were made for other potential confounders [30].

Examining the diagnosis of labor dystocia instead of the partographs is a limitation in our study. The coded diagnosis is dependent on the attending consultant at the delivery ward or at discharge and could be incomplete or misclassified. Women using LMWH during pregnancy in this study is a heterogeneous group, where a substantial part had coagulation deficiencies, possibly influencing fetal well-being. Women with LMWH are also considered high risk pregnancies, and might have had a more frequent fetal surveillance influencing actions during delivery. However, these may lead to an overestimation or underestimation of our results depending on the actions. The LMWH group was not large enough to carry out analyses stratified by underlying diagnosis for the use during pregnancy.

Another limitation of our study is that information on LMWH use was retrieved from register data on time for the dispensed drugs, and we do not have information on dose, compliance, or any information on treatment during hospital care. However there are national Swedish recommendations for use and dosage of LMWH during pregnancy [42,43]. A British prospective cohort study showed a high compliance for treatment with the LMWH enoxaparin during pregnancy [46].

The pathogenesis of VTE is multifactorial and previous thrombosis is the major risk factor for pregnancy-related VTE [19]. Several recommendations for use of LMWHs for prevention of thromboembolism have been evolved [47,48]. In Sweden, treatment with LMWHs during pregnancy is based on a risk score model where about a five-fold increased risk correspond to one point, and four points or higher risk indicates treatment with LMWH during pregnancy [43]. In the ICD-9 and -10 classification system we were not able to include all variables indicating one score point and were not able to separate different thrombophilias for underlying indication of treatment. For the group of women denoted as “others” in Table 2, we did not find any underlying recommended diagnoses for LMWH treatment in the National Patient Register. This was partly because we could not include all these minor risk factors. Other reasons could be other underlying diagnoses for treatment than recommended and that some diagnoses in the classification system were not selective enough to be included in the analyses.

Regarding presumed high dose LMWH, we used underlying diagnoses indicating high dose prophylaxis or treatment dose, which may have led to some misclassification.

Generally, women in Sweden with LMWH prophylaxis stop the use when active labor is established and next dose is administrated 4 hours after delivery. For women with high-dose prophylaxis the dose is commonly reduced to half normal prophylactic dose (i.e. dalteparin 2500 U) every 8:th to 12:th hour [42]. Use of epidural analgesia among women with prophylactic LMWH is guided by dose and time for latest injection of LMWH because of the risk of spinal/epidural hematoma and is contraindicated for women with treatment dose of LMWH. In the two previous studies [29,30] and in our study epidural analgesia was less common among women with use of LMWH during pregnancy. However, as noted in the stratified analyses, epidural analgesia did not seem to be an effect modifier in the current study.

Gestational length at delivery may be considered a mediator between LMWH and dystocia. Treatment with LMWH has been associated with preterm delivery, but this association may also be attributable to iatrogenic factors rather than spontaneous preterm deliveries [29,31–33,36]. In our study population of term and post-term births, the distribution of gestational length at delivery was similar among those with and without use of LMWH (Fig 3). We therefore allowed for adjustments of gestational age in our regression models.

The delivery is a complex, still not fully understood, process. Studies have shown that remodeling in the extracellular matrix has an important role and that the concentration of heparin sulphate proteoglycans increases during labor and could play a role in myometrial contractility and cytokines from cervical fibroblasts are involved in the ripening of the cervix [49–51]. In vitro, the LMWH dalteparin increased myometrial smooth muscle contractility and the effect was mainly observed together with oxytocin [15]. In our study we do not have information on use of oxytocin, and we could therefore not study this mediation.

In analyses where LMWH use in third trimester was divided by presumed dose, a higher risk of labor dystocia for nulliparous women was noted in the presumed high dose group compared to no treatment in adjusted analyses. These findings suggest that there might be an association between high dose LMWH use and dystocia risk in nulliparous women. However, there could be a risk of confounding by indication in this nulliparous presumed high dose group due to the high-risk profile of these pregnancies, with an increased probability of more frequent fetal surveillance influencing actions during delivery. This group does not receive epidural analgesia in the same extent due to the LMWH treatment, which also can influence actions. Hence, these results have to be interpreted with caution.

Pre-term deliveries and elective cesarean sections were excluded in our study population and thereby the study design lends itself to a potential selection bias. Hence, the study included virtually all term and post-term deliveries in Sweden with prospectively collected data and international classification of diagnoses, results could only be generalized to similar populations in developed countries.

In conclusion we could not find an association between LMWH use during pregnancy and prolonged labor when taking maternal, fetal and delivery characteristics into account. These findings are important and of general interest because there is an increased use of LMWHs during labor in developed countries. Further studies on the effects of LMWH on the labor process in other study settings are warranted.

## Ethical Approval of the Study

The study was approved by the Regional Ethical Review board No. 4 in Stockholm, Sweden: 2008/1182-31/4, date of approval September 3^rd^, 2008.

## Supporting Information

S1 Table ICD-9 and ICD-10 codes for diagnoses related to use of LMWH before and during pregnancy. (DOCX)

S2 Table Regression analysis of use of LMWH during pregnancy and labor dystocia stratified by epidural analgesia in nulliparous women with singleton infants in cephalic presentation, term or post-term births with induction or spontaneous onset in Sweden, April 2006-December 2011. (DOCX)

S3 Table Regression analysis of use of LMWH during pregnancy and labor dystocia stratified by epidural analgesia in parous women with singleton infants in cephalic presentation, term or post-term births with induction or spontaneous onset in Sweden, April 2006-December 2011. (DOCX)

S4 Table LMWH use by trimester and maternal characteristics. (DOCX)

## Abbreviations

BMI: Body mass index
LMWH: Low-molecular-weight heparin
MBR: Swedish Medical Birth Register
VTE: Venous thromboembolism
