# Supplementary material for: Does Use of Low-Molecular-Weight Heparin during Pregnancy Influence the Risk of Prolonged Labor: A Population-Based Cohort Study
Source: PLoS One. 2015 Oct 14;10(10):e0140422. doi: 10.1371/journal.pone.0140422 (PMC4605614; doi:10.1371/journal.pone.0140422)
Supplement: S1 Table — (DOCX) [file pone.0140422.s001.docx]

**Supporting information**

**Table S1. ICD-9 and ICD-10 codes for diagnoses related to use of LMWH before and during pregnancy.**

1. **Deep venous thrombosis:**

ICD-10:

O22.3 Deep phlebothrombosis in pregnancy

O22.8 Other venous complications in pregnancy

087.1 Deep phlebothrombosis in the puerperium

O87.9 Venous complications in the puerperium, unspecified

I80.1 Phlebitis and thrombophlebitis of femoral vein

I80.2 Phlebitis and thrombophlebitis of other deep vessels of lower extremities

I80.3 Phlebitis and thrombophlebitis of lower extremities, unspecified

ICD-9:

671D Deep phlebothrombosis, antepartum

671E Deep phlebothrombosis, postpartum

451B Phlebitis and thrombophlebitis, of deep vessels of lower extremities

451C Phlebitis and thrombophlebitis of lower extremities, unspecified

1. **Cerebral venous thrombosis**

ICD-10:

O22.5 Cerebral venous thrombosis in pregnancy

O87.3 Cerebral venous thrombosis in the puerperium

I63.6 Cerebral infarction due to cerebral venous thrombosis, nonpyogenic

I67.6 Nonpyogenic thrombosis of intracranial venous system

ICD-9:

671F Other phlebitis and thrombosis, cerebral venous thrombosis, thrombosis of intracranial venous sinus

437G Nonpyogenic thrombosis of intracranial venous sinus

1. **Pulmonary embolus**

ICD-10:

O88.2 Obstetric blood-clot embolism

I26 Pulmonary embolism

I27.9 Pulmonary heart disease, unspecified

ICD-9:

673C Obstetrical blood-clot embolism, puerperal pulmonary embolism NOS

415B Acute cor pulmonare, with or without infarction

416W Chronic pulmonary heart disease, unspecified, Chronic cardiopulmonary disease, chronic cor pulmonare

1. **Other thromboses/emboli**

ICD-10:

I81 Portal vein thrombosis

I82 Other venous embolism and thrombosis

I82.0 Budd-Chiari syndrome

I82.1 Thrombophlebitis migrans

I82.2 Embolism and thrombosis of vena cava

I82.3 Embolism and thrombosis of renal vein

I82.8 Embolism and thrombosis of other specified veins

I82.9 Embolism and thrombosis of unspecified vein

O08.7 Other venous complications following abortion and ectopic and molar pregnancy

ICD-9:

452 Portal vein thrombosis

453 Other venous embolism and thrombosis

453A Budd-Chiari syndrome

453B Thrombophlebitis migrans

453C Of vena cava

453D Of renal vein

453W Of other specified veins

453X Of unspecified site

1. **Atrial fibrillation**

ICD-10:

I48 Atrial fibrillation and flutter

ICD-9:

427D Atrial fibrillation and flutter

1. **Mechanical heart valve**

ICD-10:

Z95.2 Presence of prosthetic heart valve

ICD-9:

V43D Organ or tissue replaced by other means: prosthetic heart valve

1. **Primary thrombophilia**

ICD-10:

D68.5 Primary thrombophilia

Activated protein C resistance [factor V Leiden-mutation]

Deficiency: antithrombin, protein C, protein S

Prothrombin gene mutation

ICD-9: -

1. **Other Thrombophilia**

ICD-10:

D68.6 Other thrombophilia

Anticardiolipin syndrome

Antiphospholipid syndrome

Presence of the lupus anticoagulant

ICD-9: -

1. **Infertility**

ICD-10:

N97.0 Female infertility associated with anovulation

N97.1 Female infertility of tubal origin

N97.2 Female infertility of uterine origin

N97.3 Female infertility of cervical origin

N97.4 Female infertility associated with male factors

N97.8 Female infertility of other origin

N97.9 Female infertility, unspecified

Z35.0 Supervision of pregnancy with history of infertility

N98.1 Hyperstimulation of ovaries

N98.8 Other complications associated with artificial fertilization

N98.9 Complications associated with artificial fertilization, unspecified

ICD-9:

628A Infertility, female, associated with anovulation

628C Infertility, female, of tubal origin

628D Infertility, female, of uterine origin

628E Infertility, female, of cervical or vaginal origin

628W Infertility, female, of other specified origin

628X Infertility, female, of unspecified origin

V23A Pregnancy with history of infertility

1. **Recurrent pregnancy loss**

ICD-10:

N96 Habitual aborter, non-pregnant

O26.2 Pregnancy care of habitual aborter

ICD-9:

646D Habitual aborter with current pregnancy

629X Habitual aborter without current pregnancy
