# Supplementary material for: Does Use of Low-Molecular-Weight Heparin during Pregnancy Influence the Risk of Prolonged Labor: A Population-Based Cohort Study
Source: PLoS One. 2015 Oct 14;10(10):e0140422. doi: 10.1371/journal.pone.0140422 (PMC4605614; doi:10.1371/journal.pone.0140422)
Supplement: S2 Table — (DOCX) [file pone.0140422.s002.docx]

**Table S2. Regression analysis of use of LMWH during pregnancy and labor dystocia stratified by epidural analgesia in nulliparous women with singleton infants in cephalic presentation, term or post-term births with induction or spontaneous onset in Sweden, April 2006-December 2011.**

|  | **Labor dystocia** | | | | | | | |
| --- | --- | --- | --- | --- | --- | --- | --- | --- |
|  | **Nulliparous women** | | | | | | | |
|  | **Without epidural**  **(N= 115 960)** | | | | | | | |
| **Groups of treatment with LMWH** | **N total** | **%** | **OR** | **(95% CI)** | **aOR* mod**  **1** | **(95% CI)** | **aOR****  **mod**  **2** | **(95% CI)** |
| **No treatment** | 114 760 | 11.2 | 1.00 | - | 1.00 | (-) | 1.00 | (-) |
| **Third trimester** | 844 | 12.1 | 1.09 | (0.89-1.34) | 1.00 | (0.80-1.24) | 1.03 | (0.82-1.28) |
| **First and /or second trimester** | 356 | 15.2 | 1.42 | (1.06-1.89) | 1.21 | (0.89-1.65) | 1.25 | (0.91-1.71) |
|  | **With epidural**  **(N=116 144)** | | | | | | | |
| **No treatment** | 115 215 | 31.1 | 1.00 | (-) | 1.00 | (-) | 1.00 | (-) |
| **Third trimester** | 598 | 30.9 | 0.99 | (0.83-1.18) | 0.95 | (0.80-1.15) | 0.98 | (0.82-1.18) |
| **First and /or second trimester** | 331 | 33.2 | 1.10 | (0.88-1.39) | 0.98 | (0.77-1.24) | 0.99 | (0.78-1.27) |

* Model 1: Adjustments for maternal characteristics: treatment with LMWH, age, height, BMI, smoking during pregnancy, education, diabetes, hypertensive disease, assisted reproduction, year of birth and onset of labour.

** Model 2: Adjustments for characteristics in model 1 and gestational length at birth.
