# Supplementary material for: Does Use of Low-Molecular-Weight Heparin during Pregnancy Influence the Risk of Prolonged Labor: A Population-Based Cohort Study
Source: PLoS One. 2015 Oct 14;10(10):e0140422. doi: 10.1371/journal.pone.0140422 (PMC4605614; doi:10.1371/journal.pone.0140422)
Supplement: S3 Table — (DOCX) [file pone.0140422.s003.docx]

**Table S3. Regression analysis of use of LMWH during pregnancy and labor dystocia stratified by epidural analgesia in parous women with singleton infants in cephalic presentation, term or post-term births with induction or spontaneous onset in Sweden, April 2006-December 2011.**

|  | **Labor dystocia** | | | | | | | |
| --- | --- | --- | --- | --- | --- | --- | --- | --- |
|  | **Parous women** | | | | | | | |
|  | **Without epidural**  **(N= 230 390)** | | | | | | | |
| **Groups of treatment with LMWH** | **N total** | **%** | **OR** | **(95% CI)** | **aOR* mod**  **1** | **(95% CI)** | **aOR****  **mod**  **2** | **(95% CI)** |
| **No treatment** | 227 806 | 2.6 | 1.00 | (-) | 1.00 | (-) | 1.00 | (-) |
| **Third trimester** | 2 025 | 2.7 | 1.04 | (0.79-1.36) | 0.93 | (0.69-1.24) | 0.96 | (0.72-1.28) |
| **First and /or second trimester** | 559 | 2.9 | 1.11 | (0.68-1.83) | 0.95 | (0.55-1.62) | 0.99 | (0.58-1.69) |
|  | **With epidural**  **(N=52 381)** | | | | | | | |
| **No treatment** | 51 819 | 13.8 | 1.00 | (-) | 1.00 | (-) | 1.00 | (-) |
| **Third trimester** | 393 | 12.7 | 0.91 | (0.68-1.23) | 0.93 | (0.68-1.27) | 0.99 | (0.72-1.35) |
| **First and /or second trimester** | 169 | 14.2 | 1.04 | (0.67-1.60) | 0.88 | (0.55-1.40) | 0.94 | (0.58-1.51) |

* Model 1: Adjustments for maternal characteristics: treatment with LMWH, age, height, BMI, smoking during pregnancy, diabetes, hypertensive disease, assisted reproduction, education, year of birth and onset of labor.

** Model 2: Adjustments for characteristics in model 1, and gestational length at birth.
