# Supplementary material for: Does Use of Low-Molecular-Weight Heparin during Pregnancy Influence the Risk of Prolonged Labor: A Population-Based Cohort Study
Source: PLoS One. 2015 Oct 14;10(10):e0140422. doi: 10.1371/journal.pone.0140422 (PMC4605614; doi:10.1371/journal.pone.0140422)
Supplement: S4 Table — (DOCX) [file pone.0140422.s004.docx]

**Table S4:** **LMWH use by trimester and maternal characteristics.**

|  | **Nulliparous women** | | | | | **Parous women** | | | |
| --- | --- | --- | --- | --- | --- | --- | --- | --- | --- |
|  |  | | **Use of LMWH** | | |  | | **Use of LMWH** | |
| **Maternal characteristics** | **N Total** | **Third trimester**  **(%)** | | **First and/or second trimester**  **(%)** | **N Total** | | **Third trimester**  **(%)** | | **First and/or second**  **trimester**  **(%)** |
|  |  |  | |  |  | |  | |  |
| **Labor dystocia** |  |  | |  |  | |  | |  |
| **No** | 182 921 | 0.63 | | 0.29 | 269 626 | | 0.86 | | 0.26 |
| **Yes** | 49 183 | 0.58 | | 0.33 | 13 145 | | 0.79 | | 0.30 |
|  |  |  | |  |  | |  | |  |
| **Age (years)** |  |  | |  |  | |  | |  |
| ≤ 29* | 139 307 | 0.45 | | 0.16 | 91 152 | | 0.63 | | 0.17 |
| 30-34 | 67 135 | 0.76 | | 0.40 | 111 556 | | 0.84 | | 0.22 |
| 35-39 | 21 972 | 1.13 | | 0.72 | 66 404 | | 1.06 | | 0.37 |
| ≥ 40 | 3 690 | 1.60 | | 1.14 | 13 659 | | 1.52 | | 0.59 |
|  |  |  | |  |  | |  | |  |
| **Height (cm)** |  |  | |  |  | |  | |  |
| ≤ 154 | 6 384 | 0.30 | | 0.14 | 8 771 | | 0.38 | | 0.21 |
| 155-164 | 75 714 | 0.47 | | 0.25 | 95 134 | | 0.71 | | 0.23 |
| 165-174* | 115 347 | 0.70 | | 0.34 | 139 039 | | 0.95 | | 0.27 |
| ≥ 175 | 23 929 | 0.79 | | 0.33 | 27 176 | | 1.11 | | 0.33 |
| **Missing** | 10 730 |  | |  | 12 651 | |  | |  |
|  |  |  | |  |  | |  | |  |
| **BMI ((kg/m^2^)** |  |  | |  |  | |  | |  |
| < 18.5 | 6 113 | 0.51 | | 0.26 | 4 984 | | 0.74 | | 0.18 |
| 18.5-24.9* | 139 130 | 0.54 | | 0.30 | 150 881 | | 0.75 | | 0.24 |
| 25.0-29.9 | 48 270 | 0.72 | | 0.30 | 70 628 | | 0.89 | | 0.28 |
| 30.0-34.9 | 14 435 | 0.84 | | 0.39 | 24 427 | | 1.19 | | 0.31 |
| ≥ 35 | 5 821 | 1.37 | | 0.36 | 10 064 | | 1.49 | | 0.40 |
| Missing | 18 335 |  | |  | 21 787 | |  | |  |
|  |  |  | |  |  | |  | |  |
| **Smoking during pregnancy** |  |  | |  |  | |  | |  |
| Yes | 16 435 | 0.64 | | 0.17 | 20 341 | | 1.06 | | 0.29 |
| No* | 207 649 | 0.62 | | 0.31 | 253 030 | | 0.84 | | 0.26 |
| Missing | 8 020 |  | |  | 9 400 | |  | |  |
|  |  |  | |  |  | |  | |  |
| **Diabetes** |  |  | |  |  | |  | |  |
| No* | 229 461 | 0.62 | | 0.29 | 278 690 | | 0.85 | | 0.26 |
| Gestational | 1 924 | 0.94 | | 0.47 | 3 171 | | 0.98 | | 0.28 |
| Pre-gestational | 719 | 0.97 | | 0.28 | 910 | | 1.10 | | 0.55 |
|  |  |  | |  |  | |  | |  |
| **Hypertensive disease** |  |  | |  |  | |  | |  |
| No* | 222 954 | 0.62 | | 0.29 | 277 475 | | 0.84 | | 0.25 |
| Chronic | 1 293 | 1.47 | | 0.46 | 1 906 | | 1.36 | | 0.47 |
| Preeclampsia | 7 857 | 0.64 | | 0.37 | 3 390 | | 1.45 | | 0.56 |
|  |  |  | |  |  | |  | |  |
| **Assisted reproduction** |  |  | |  |  | |  | |  |
| No* | 217 422 | 0.58 | | 0.14 | 275 769 | | 0.85 | | 0.22 |
| In vitro fertilization | 110 674 | 1.29 | | 2.93 | 4 901 | | 1.35 | | 2.20 |
| Ovulation stimulation | 4 008 | 0.90 | | 1.50 | 2 101 | | 0.90 | | 1.24 |
|  |  |  | |  |  | |  | |  |
| **Education** |  |  | |  |  | |  | |  |
| Years of formal education ≤12* | 102 314 | 0.58 | | 0.24 | 138 447 | | 0.84 | | 0.27 |
| Years of formal education >12 | 123 417 | 0.68 | | 0.35 | 137 956 | | 0.88 | | 0.25 |
| Missing | 6 373 |  | |  | 6 368 | |  | |  |
|  |  |  | |  |  | |  | |  |
| **Fetal and delivery characteristics** | **N Total** | **Third trimester**  **(%)** | | **First and/or second trimester**  **(%)** | **N Total** | | **Third trimester**  **(%)** | | **First and/or second**  **trimester**  **(%)** |
| **Gestational length at birth (weeks)** |  |  | |  |  | |  | |  |
| 37 | 10 807 | 0.78 | | 0.33 | 12 363 | | 1.27 | | 0.57 |
| 38 | 24 943 | 0.70 | | 0.32 | 33 908 | | 1.14 | | 0.32 |
| 39 | 52 468 | 0.59 | | 0.30 | 72 345 | | 0.85 | | 0.27 |
| 40* | 71 508 | 0.62 | | 0.30 | 92 468 | | 0.74 | | 0.22 |
| 41 | 50 375 | 0.64 | | 0.28 | 54 455 | | 0.80 | | 0.22 |
| ≥ 42 | 21 927 | 0.49 | | 0.25 | 17 102 | | 0.78 | | 0.16 |
| Missing | 76 |  | |  | 130 | |  | |  |
|  |  |  | |  |  | |  | |  |
| **Onset of delivery** |  |  | |  |  | |  | |  |
| Spontaneous | 197 122 | 0.55 | | 0.27 | 246 334 | | 0.75 | | 0.23 |
| Induction | 34 057 | 1.00 | | 0.45 | 35 188 | | 1.61 | | 0.46 |
| Missing | 925 |  | |  | 1 249 | |  | |  |
|  |  |  | |  |  | |  | |  |
| **Epidural** |  |  | |  |  | |  | |  |
| No | 115 960 | 0.73 | | 0.31 | 230 390 | | 0.88 | | 0.24 |
| Yes | 116 144 | 0.51 | | 0.28 | 52 381 | | 0.75 | | 0.32 |
|  |  |  | |  |  | |  | |  |
| **Birth weight (grams)** |  |  | |  |  | |  | |  |
| < 3000 | 28 932 | 0.67 | | 0.30 | 21 000 | | 1.17 | | 0.37 |
| 3000-3499* | 85 649 | 0.66 | | 0.30 | 83 197 | | 0.85 | | 0.28 |
| 3500-3999 | 82 233 | 0.58 | | 0.31 | 110 473 | | 0.85 | | 0.25 |
| 4000-4499 | 29 339 | 0.59 | | 0.25 | 54 054 | | 0.79 | | 0.22 |
| ≥4500 | 5 624 | 0.55 | | 0.30 | 13 747 | | 0.65 | | 0.15 |
| Missing | 327 |  | |  | 300 | |  | |  |
